# Commentary: The Tumor Markers and Blood Inflammation Markers Are More Likely to Be the Indicators for Differentiating Benign and Malignant Pancreatic Mucinous Cystic Neoplasms

**DOI:** 10.3389/fonc.2022.901010

**Published:** 2022-06-02

**Authors:** Jiang Liu, Ying Xing, Liqi Sun

**Affiliations:** ^1^ Department of Gastroenterology, Huzhou Hospital of Zhejiang University, Affiliated Central Hospital Huzhou University, Huzhou, China; ^2^ Department of Gastroenterology, 72nd Group Army Hospital, Huzhou University, Huzhou, China

**Keywords:** mucinous cystic neoplasm (MCN), serous cystic neoplasm, serum tumor markers, blood inflammation markers, EUS – endoscopic ultrasonography

To the editor:

We read with great interest the article by Wang et al. (1) which identified the value of carbohydrate antigen 19-9 (CA19-9) and [lymphocyte × albumin (ALB), LA] in differentiating serous cystic neoplasms (SCNs) and mucinous cystic neoplasms (MCNs). We appreciated the great efforts that the authors made to help the management of pancreatic cystic neoplasms (PCNs). However, some concerns arise which may limit the strength of the evidence level of this study.

First, it is widely acknowledged that a high level of CA19-9 is associated with advanced PCNs. The value of inflammatory markers based on circulating blood cells in predicting advanced PCNs is not well studied. The hypothesis that CA19-9 and inflammatory markers may be useful in differentiating MCN and SCN raised by the authors is mostly based on studies that focus on pancreatic cancer and other malignant tumors. However, MCNs are premalignant tumors, only 10%–17% of which will harbor malignancy (2). The hypothesis may not be applicable to PCN due to lack of reference support. Moreover, the cyst fluid CA19-9 has little value in distinguishing mucinous and non-mucinous PCNs and even the value of cyst fluid carcinoma embryonic antigen (CEA) was doubted at present (2). The conclusions are opposite to the statement in the introduction section raised by authors.

Second, eight MCNs were reported to have a malignant transformation in the study cohort. It means that the MCN patients had a more malignant rate than SCNs (18.8% vs 0%, P = 0.008). Hence, the results of the study merely indicate that CA19-9 and LA may be useful in differentiating benign and malignant tumors. The eight cases with malignancy should be excluded to further identify whether these indicators are still useful in differentiating MCNs and SCNs.

Third, the lymphocyte and ALB change greatly due to food intake and activity, even in the morning and evening of the day. At what time lymphocyte and ALB are tested should be clearly clarified. The analysis of LA is meaningless if they were evaluated at various stages of diseases (preoperative or postoperative, early tumor stage or later tumor stage, etc.). To avoid bias, each patient’s nutritional state should be further assessed, too.

Fourth, the value of cytology obtained by endoscopic ultrasound-guided fine-needle aspiration (EUS-FNA) is limited in differentiating MCNs and SCNs with 42% sensitivity (3). Only about 35% of cytologic samples obtained by EUS-FNA were informative (4). The reported accuracy of EUS morphology alone for differentiating mucinous from non-mucinous PCNs is relatively low (48%–94%) (5). Although cytology obtained by EUS-FNA is highly specific (83%–100%), it is relatively insensitive (27–48%) (5). In general, the value of EUS-FNA in making a specific subtype of PCNs is limited. We noticed in Supplementary Table 2 that six SCN patients underwent EUS-FNA alone to make a final diagnosis. SCN was one of the non-mucinous PCN types. So, what are the diagnostic criteria of SCN without cytology? Imaging diagnoses are not definite diagnoses.

Fifth, only eight patients with malignant MCNs in this study were correctly resected. Even the patients who had a low level of cyst fluid CEA underwent surgery. The indications for surgery should be clarified.

Sixth, the authors stated that “The results of the ROC curve showed that the AUC values of serum CA199 and LA in differentiating SCNs and MCNs were 0.6734 and 0.6765, respectively, indicating that they have a certain value in the differential diagnosis of SCNs and MCN”. However, with AUC <0.7, we may not be able to say the indicators had certain values.

Seventh, MCNs and mucinous ovarian tumors both originate from primordial germ cells, and ovarian-like stroma can be obtained from the tissue of MCNs (6); thus, MCNs may have an increased CA125 level as well as ovarian tumors. CA125 may be a better indicator than CA19-9 in differentiating SCNs and MCNs (7).

In general, the study provided a new thought to help us understand MCNs and SCNs better. However, great bias may exist in this study and the concerns prevent the study from making strong conclusions. The predictive efficacy of CA199 and LA still needs to be verified in future clinical work.

## Author Contributions

LS was responsible for the study design/planning, study conduct, and revision of the paper. JL wrote this paper. YX assisted in writing and revising the paper. All authors contributed to the article and approved the submitted version.

## Conflict of Interest

The authors declare that the research was conducted in the absence of any commercial or financial relationships that could be construed as a potential conflict of interest.

## Publisher’s Note

All claims expressed in this article are solely those of the authors and do not necessarily represent those of their affiliated organizations, or those of the publisher, the editors and the reviewers. Any product that may be evaluated in this article, or claim that may be made by its manufacturer, is not guaranteed or endorsed by the publisher.
